# Building a Practice-Based Research Agenda for Wildfire Smoke and Health: A Report of the 2018 Washington Wildfire Smoke Risk Communication Stakeholder Synthesis Symposium

**DOI:** 10.3390/ijerph16132398

**Published:** 2019-07-06

**Authors:** Nicole A. Errett, Heidi A. Roop, Claire Pendergrast, C. Bradley Kramer, Annie Doubleday, Kim Anh Tran, Tania M. Busch Isaksen

**Affiliations:** 1 Department of Environmental and Occupational Health Sciences, University of Washington School of Public Health, Seattle, WA 98195, USA; 2 Department of Health Services, University of Washington School of Public Health, Seattle, WA 98195, USA; 3 Climate Impacts Group, University of Washington, Seattle, WA 98195, USA

**Keywords:** wildfire smoke, risk communication, risk management, public health practice, research needs

## Abstract

*Background*: As climate change is expected to result in more frequent, larger fires and associated smoke impacts, creating and sustaining wildfire smoke-resilient communities is an urgent public health priority. Following two summers of persistent and extreme wildfire smoke events in Washington state, the need for additional research on wildfire smoke health impacts, risk communication, and risk reduction, and an associated greater coordination between researcher and practitioner communities, is of paramount importance. *Objectives:* On 30 October 2018, the University of Washington hosted a Wildfire Smoke Risk Communication Stakeholder Synthesis Symposium in Seattle, Washington. The goals of the symposium were to identify and prioritize practice-based information gaps necessary to promote effective wildfire smoke risk communication and risk reduction across Washington state, foster collaboration among practitioners and academics to address information gaps using research, and provide regional stakeholders with access to the best available health and climate science about current and future wildfire risks. *Methods*: Seventy-six Washington state practitioners and academics with relevant professional responsibilities or expertise in wildfire smoke and health engaged in small group discussions using the “World Café Method” to identify practice-relevant research needs related to wildfire smoke and health. Notes from each discussion were coded and qualitatively analyzed using a content analysis approach. *Discussion*: Washington state’s public health and air quality practitioners need additional evidence to communicate and reduce wildfire smoke risk. Exposure, health risk, risk communication, behavior change and interventions, and legal and policy research needs were identified, along with the need to develop research infrastructure to support wildfire smoke and health science. Practice-relevant, collaborative research should be prioritized to address this increasing health threat.

## 1. Introduction

Creating and sustaining wildfire smoke-resilient communities is an urgent public health priority, and one that is of high concern for Washington state agencies involved in wildfire smoke risk reduction.

For the past several years, the western part of the United States has experienced extensive and damaging wildfire seasons [1]. Instances of wildfires burning in locations previously thought of as low-risk, and significant smoke permeating dense urban areas, have been a wakeup call to communities with little or no experience dealing with wildfires and/or extended exposure to wildfire smoke. In Washington state, the past two summers have been particularly impactful on urban residents not used to summertime burns. In 2017, fires in Western Washington resulted in moderate to unhealthy air quality for over 2 weeks, while Eastern Washington experienced 15 days with a range of poor air from “unhealthy for sensitive groups” up to “very unhealthy for all” [2]. At the same time, the state experienced higher than average summertime temperatures. The combination of smoke and high heat presented a unique challenge for densely populated urban areas in Western Washington, where air conditioning is limited in many residential and commercial buildings. In 2018, smoke returned to dominate the normally enjoyable August weather, impacting summertime activities and ushering in news coverage like “Air quality around the Seattle region varied from unhealthy to very unhealthy on Wednesday, with index readings far worse than those at the same time in Bejing [sic], China, infamous for its polluted skies” [3]. By the end of the summer, Washington state residents had experienced 42 days where at least 1 federal air monitor had measured PM2.5 in the *unhealthy for sensitive groups* or worse air quality categories [2].

There is growing evidence suggesting that wildfire smoke poses a serious public health threat. Wildfire smoke has been shown to be associated with respiratory morbidity and all-cause mortality [4,5,6]. Mixed evidence has pointed to a possible association with cardiovascular health impacts [4,5,6]. Yet, uncertainties remain. For instance, the need for research in wider geographical areas over longer periods of time to further understand adverse health outcome risks and sensitive groups, as well as identify social and contextual determinants of the exposure–health outcome relationship, has been discussed [6]. Amidst widespread exposure, public health practitioners and others serving impacted communities are grappling with assessing, communicating, and managing risk with insufficient evidence.

The risks of wildfire smoke are compounded by strong evidence that climate change is bringing more and bigger wildfires to Washington state and the greater western United States (e.g., [7,8,9,10]). Human-caused climate change is estimated to have led to an additional 4.2 million hectares of area burned between 1984–2015 across the western U.S.; this is nearly double the area burned expected in the absence of human-caused climate change [7]. Climate change is also leading to increased frequency, duration, and intensity of wildfires [11,12,13,14], and longer regional fire seasons [8,10,11]. Further, future projections of wildfire in the Northwest suggest that the area burned by wildfires by the 2080s could more than double relative to 1916–2006 (under a medium emissions scenario) [9].

As climate change alters the pattern of wildfires across the Northwest, the implications for the extent and presence of wildfire smoke remain an active area of research for public health and climate researchers alike. Following two summers of intense wildfire smoke across Washington state, it is clear that there is the need for more research on wildfire smoke risk, future wildfire smoke extent and duration, and the human health implications of wildfire smoke. There is also a need for greater coordination between researcher and practitioner communities in order to develop and deploy adaptations, solutions, and preventative actions that minimize the risks associated with wildfire and wildfire smoke in a changing climate.

In an effort to respond to these needs and to engage regional stakeholders in dialogue around the risks and responses to wildfire smoke, the Wildfire Smoke Risk Communication Stakeholder Synthesis Symposium was held in Seattle, Washington on 30 October 2018. The goals of the symposium were to identify and prioritize practice-based information gaps necessary to: Promote effective wildfire smoke risk communication and risk reduction across Washington state; foster collaboration among practitioners and academics to address information gaps using research; and provide regional stakeholders with access to the best available climate science about current and future wildfire risks.

Here, we present the information gaps and research priorities identified during this interactive workshop in order to guide the development of a practitioner-relevant research agenda, with the ultimate goal of helping Washington state communities, agencies, researchers, and practitioners reduce community-wide risks from exposure to wildfire smoke.

## 2. Methods

The symposium was designed to provide participants with opportunities to hear about the state of the science, engage in discussions around the real-world challenges of addressing wildfire smoke risk in communities, and to actively share their knowledge and expertise to help guide the development of a research agenda for future work. The symposium was coordinated by an interdisciplinary team of University of Washington faculty and students, and advertised through listservs and/or social media maintained by the University of Washington’s Department of Environmental and Occupational Health Sciences, Program on Climate Change, Climate Impacts Group, the Northwest Climate Adaptation Science Center, and the Northwest Center for Public Health Practice. Additional promotion was achieved through faculty and students’ practice-based networks and collaborators. Of over 117 interested participants, a purposefully selected group of 76 regional stakeholders (including the organizing team and facilitators/notetakers) representing over 30 agencies, organizations, communities, and tribes attended the symposium (Table 1). Participants were selected to ensure a diverse representation of organizations and roles in assessing, communicating, managing, and/or researching wildfire smoke risk across Washington state. Symposium registration provided information about participants’ goals and interests, and the planning team aligned the content of the workshop to meet the needs and desired learning outcomes of the audience.

The symposium began with presentations on the health risks of wildfire smoke, the connection between wildfire smoke and climate change, and examples of ongoing research efforts to better characterize wildfire smoke impacts on health to inform disaster recovery and public health practice. Participants also heard from a panel of practitioners from across the state who discussed their experiences, challenges, and needs related to wildfire smoke events. Following these presentations and panel discussions, participants engaged in small group discussions using the “World Café Method,” a technique designed to foster large group dialogue that is deliberate in its appreciation of local knowledge [15]. Eight tables were divided into four different subpopulations (two tables each): (1) Workers, (2) at-risk/susceptible populations, (3) rural communities, and (4) urban/suburban communities. Participants engaged in four 20-min interactive group discussions. At the end of each 20-min discussion, participants rotated to form new groups at different tables. Each 20-min discussion focused on the following overarching questions:

1. Who is uniquely susceptible to wildfire smoke in this community? Why?
2. How can we effectively communicate risk to this population?
3. How can we reduce risk to this population?
4. How can research improve preparedness and response to future wildfire smoke events?

Eight notetakers and eight facilitators—one each for each table during each round of discussion—facilitated and documented the discussions. Facilitators were trained on the use of a discussion guide that included prompts and additional questions to ensure a robust discussion during each round. Facilitators remained at each table and provided a summary of prior discussions to each incoming group to build on. A room facilitator walked the group through the process and the overarching goals for each round of discussion.

Facilitators captured the discussion on a flip chart while notetakers documented each conversation. Participants were encouraged to write their ideas on post-it notes that were pinned on the flip chart. Tables were also draped with butcher paper and participants were provided with markers and note cards to organize ideas and promote creative energy and engagement across different personality types and learning styles. Participants were also provided the opportunity to contribute to discussions about other subpopulations by writing and submitting comments on index cards. Facilitators reported out the identified research needs and participants had the opportunity to clarify or add to the needs discussed. Notetakers at each table documented conversations during each discussion. Facilitators reviewed and edited notes for accuracy and completeness, and to ensure that they reflected notes captured on other media. After the final café session, table facilitators and notetakers met and synthesized the research needs discussed at their tables for each of the four subpopulations represented.

Qualitative content analysis methods were used to identify and synthesize research needs documented in the notes [16]. Notes were first reviewed in their entirety to identify broad categories of research needs discussed. These broad categories of research needs were institutionalized into a codebook, wherein each category identified in the data familiarization process became a code with a code definition and examples of when to apply the code (NAE). A second member of the research team (TBI) reviewed the codebook to ensure that it was clear and comprehensive. A team-based, consensus-building coding approach was then employed wherein two coders (NAE and TBI) worked together to discuss and apply codes to qualitative data using NVivo qualitative data analysis software (QSR International Pty Ltd. Version 12, 2018). Coded text was reviewed to identify key themes and contextual considerations by research category (NAE). Analytic memos were then developed (NAE) for each code to summarize the research needs that emerged across the discussions. All members of the author team had also served as facilitators or note-takers during the symposium. They reviewed the findings outlined in the analytic memos to confirm that the research needs presented were reflective of the world café discussions, and that minor points and contextual factors were clearly and consistently considered.

This research was determined to be human subjects research that qualifies for exempt status (category 2) by the University of Washington Human Subjects Division.

## 3. Results and Discussion

**Figure 1:**
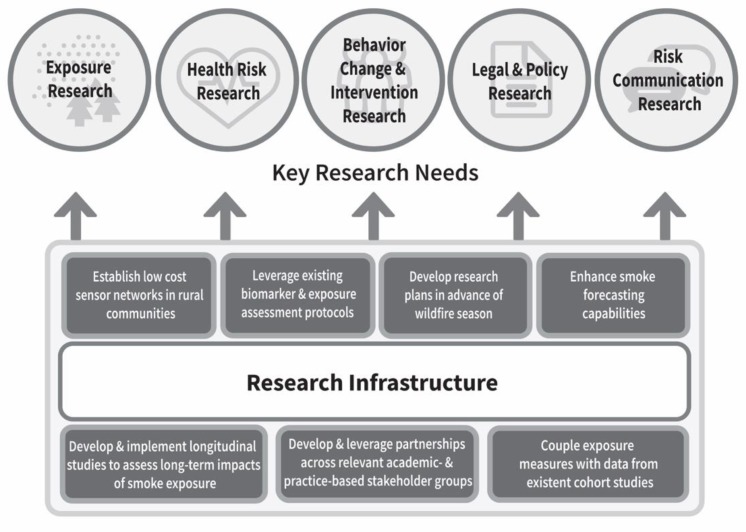
Wildfire smoke research infrastructure needs.

Participants identified research needs that were grouped into the following research topics: Exposure science, health risk research, risk communication research, behavior change and interventions research, and legal and policy research. Key research needs by topic are highlighted in Table 2 and described in detail below. Research infrastructure needs identified are also discussed (Figure 1), along with a summary of the authors’ recommendations for the next steps. Notably, the order of research topics and needs presented herein are not in any way reflective of participant prioritization of research needs.

The identified research needs are by no means inclusive of all research needs but reflect areas that can generate evidence deemed necessary to participating stakeholders responsible for communicating or managing wildfire smoke risk in Washington state. We suggest additional engagement with practitioners and academics outside of Washington state, and possibly internationally, to further inform a robust, practice-based research agenda.

The sheer interest in our symposium demonstrates the salience of this public health issue to practitioner and academic communities. We hope that this report can galvanize stakeholders to work collaboratively to address this important public health issue. We purposefully employed a community-based participatory research approach, designed to leverage and build on community strengths and address locally relevant issues and concerns, balance the development of resilience science and practice, facilitate co-learning and community resilience capacity development, and ensure a collaborative, equitable partnership that empowers community stakeholders [17]. We believe that this approach can be replicated to address other emerging environmental health issues to inform the development of practice-based research agendas and trusted relationships. We encourage researchers that choose to conduct research on these topics to build relationships with community partners and maintain a meaningful and equitable partnership throughout the process to ensure research remains practice-relevant and affects change.

**Exposure Research:** Participants called for more robust research to quantify and qualify wildfire smoke exposure. Participants described a need to identify the amount of wildfire smoke people are being exposed to. They identified the need for a definition of the hazard or “smoke wave” to define and communicate risk.

Research on how exposure levels (including PM2.5 and Volatile Organic Compounds (VOCs)) change by geographic distance from the wildfire itself was identified as a need. It was noted that additional research is necessary on whether particles change form or size, or adhere to other compounds, as they travel. In addition, participants described research needs related to pesticide and wildfire smoke co-exposures, and how their interaction impacts exposures. For instance, they questioned whether wildfire smoke particulates increased exposure to pesticides by acting as a deposition point, or if they acted to sequester the pesticides.

Gaps in information on the quality of indoor air during wildfire events were noted. Participants questioned whether indoor air quality was better than outdoor air quality, especially in leaky homes. They noted that indoor air monitoring was necessary to evaluate the effectiveness of interventions (e.g., air filters) at improving air quality. They suggested selecting a community as a case study to quantify indoor exposures during smoke events compared to exposures experienced throughout the rest of the year. Car (in-cabin) air exposure assessments were also identified as a need.

Participants noted that significant research has been done on the composition of traffic and cigarette smoke, but pointed out that more information was necessary on wood smoke and wildfire smoke specifically. They suggested exploring what could be learned from cookstove research in low- income countries.

**Health Risk Research:** The need to assess the health impacts of acute (short-term) and chronic (long-term) exposure, as well as long-term impacts of acute, chronic, and cumulative exposure, was also discussed. Gaps in understanding the long-term impacts of exposure and of childhood exposure were identified. Participants questioned whether breaks in exposure have significant impacts on risk.

Participants emphasized a need to better understand the health risks for particular subpopulations (e.g., children, healthy people, sensitive populations, workers, firefighters, etc.). They described the need to assess health impacts of worker exposure in terms of cumulative exposure (including from side jobs) and total worker health. Participants also called for research on specific health outcomes and on the impact of particular health conditions on risk from wildfire smoke exposure. The need to identify the health impacts of exposure to both VOCs and PM2.5 was discussed.

Finally, participants described the need to quantify the relative risks of exposure across different levels (e.g., “healthy” versus “unhealthy”) of air quality indices, such as the Environmental Protection Agency’s Air Quality Index (AQI) and Washington’s Air Quality Advisory (WAQA).

**Risk Communication Research:** Participants described the need for better risk-related data to inform risk communication strategies, including information related to compounding exposures (e.g., wildfire smoke and heat). As stated above, participants identified the need to define the hazard (i.e., what is a “wildfire smoke event” or “smoke wave”) to inform and trigger risk communication.

Participants described the need for research to better understand risk awareness and perception, particularly among sensitive populations, as well as associated educational needs. Participants acknowledged the need for targeted communications to different subpopulations (e.g., workers, rural communities, homeless populations), and called for additional research to inform risk communication strategies and consistent messaging by assessing underlying values, motivators, and networks/trusted messengers among subpopulations. Community-level asset mapping was identified as an opportunity to identify trusted communication channels and messengers in advance of the next smoke event.

Participants identified a need for research to evaluate the effectiveness of specific messages (e.g., health outcomes emphasized and interventions specified) and mechanisms of communication by subpopulation on self-efficacy and behavior change. They noted that certain interventions are impractical for certain populations (e.g., telling outdoor workers to stay inside) and discussed associated needs to identify messages that would resonate with particular subpopulations. They discussed the need to assess the effectiveness of using analogies (e.g., campfires, cigarettes, exposure in other polluted cities) to communicate risk; for example, a message comparing wildfire smoke to cigarettes smoked may not resonate with a smoker. Participants noted that there is a particular need for research about how to effectively communicate risk to populations resistant to, or in denial of, messaging related to wildfire smoke impacts on health. They also acknowledged the need for research on how local governments should address fear, outrage, and anxiety in their messaging around wildfire smoke.

Participants called for additional guidance and information on how to communicate inconsistencies across air quality indices. For example, guidance and indices issued by the Environmental Protection Agency, Washington’s Department of Ecology, Occupational Safety and Health Administration, and Washington’s Department of Labor and Industries all have different definitions of what is “unhealthy” or “unsafe”.

Finally, participants called for evaluation of risk communication activities conducted during previous wildfire smoke events, including the effectiveness of specific messages delivered and the reach of communication mechanisms utilized (e.g., radio, print, social media).

**Behavior Change and Intervention Research:** Participants identified a variety of research needs related to the effectiveness, sufficiency, and feasibility of different individual- and community-level interventions, including workplace controls. They also identified a need to assess the effectiveness of behavior changes to minimize risk. Again, participants identified the need for better risk-related data to inform and assess risk reduction strategies.

Participants called for more research on how people are currently responding to smoke events. They wanted a better understanding of which interventions people are using, how they are using them, and what their current behaviors are during smoke events.

A recurring theme across the discussions was the need for research on N-95 masks. N-95 masks are distributed to the public by several Washington jurisdictions during smoke events without fit testing. Participants called for testing on whether or not the masks distributed are effective and properly fitted. They questioned whether or not video tutorials could be effective compared to fit testing in promoting an effective fit. Participants called for research on the effectiveness of mask use among children, and on the relative effectiveness of masks with different designs. They also called for research on the conditions during which mask use is effective, including during different levels of exposure and during extreme heat events. They identified the need for information on the lifespan of a mask, both in use and in storage.

Amidst long-term and compounding exposure concerns, participants called for research on the effectiveness and sufficiency of interventions, e.g., ensuring that air quality in sleeping environments is high quality, taking breaks in filtered cars during the workday, and evacuation of pregnant women. Participants also identified needs to determine the benefits of building clean air shelters or making building code changes. They questioned the risk–benefit of air conditioning.

Participants noted that taking certain actions might not be feasible for particular populations, (e.g., staying indoors for homeless or outdoor workers). Participants described the need for simple, low-cost interventions and called for research to identify higher risk and lower risk activities to inform population-specific interventions. They also noted that such research could allow populations to have choices, to take advantage of daily variability in exposure (e.g., by performing a high-risk task at a time of the day when smoke exposure is lower) and to inform when mitigation activities (e.g., not driving a plow during extremely high smoke levels) might be appropriate even if risk reduction activities are not feasible. Participants also called for research on economic thresholds or tipping points for taking action. In other words, they described a need to understand if particular price points (e.g., of a clean air filter) prohibited individuals from choosing to use an intervention.

Beyond protection from the physical health impacts of wildfire smoke, participants called for research on interventions designed to promote well-being and happiness and reduce feelings of helplessness, as well as self-care to reduce stress and anxiety.

**Legal and Policy Research:** Participants called for additional data to inform and evaluate policy solutions intended to minimize the risk of wildfire smoke. Participants described the challenges wildfire smoke presents in occupational health and safety (OHS) regulations. Typically, OHS regulations are based on exposures over an 8-h workday for a healthy worker. While they described gaps in regulations for short-term exposures, they also noted the challenges in regulating wildfire smoke exposure, for which exposure is likely to be outside of the employer’s control and extend beyond work hours. Participants described the need for clarity on situations where OHS regulations apply to exposure control. They identified policy research needs related to authority, appropriateness, and feasibility of using regulation to control exposure to this environmental health hazard.

Participants also identified needs for specific science-based exposure limits for outdoor workers and for indoor air. Participants emphasized that rule-making and decision making must account for economic impacts. In addition to regulatory solutions, a need to identify best management practices for employees was described. They described the lack of evidence-based recommendations for workers and the public at different levels of exposure. They noted that employers should identify and ensure special protections are made for at-risk workers but questioned how to do that without promoting bias against these at-risk workers.

Participants identified questions related to liability and workers compensation solutions, such as “Should there be a program or other mechanism to compensate workers for symptoms related to exposure?” They noted that the impacts on seasonal and migrant workers are not tracked and questioned who should be held responsible for impacts experienced by these populations.

Participants called for health impact assessments of building code changes and other interventions. They also described a need to document the health impacts and healthcare costs of wildfire smoke exposure-related impacts to inform policy and quantify the economic impacts. They questioned how government agencies should balance health and economic estimates when making decisions (e.g., local governments calling for the closure of major outdoor events). Participants called for research on international policy and practice solutions to wildfire smoke risk; for example, “What are countries like Russia and Australia doing to address smoke exposure?”

**Research Infrastructure Needs:** Participants identified research infrastructure needs to support wildfire smoke and health science (Figure 1). They called for infrastructure to assess long-term impacts of smoke exposure. Specifically, they identified needs for longitudinal studies and associated methods. They identified the presence of good protocols to measure biomarkers and exposure that could be leveraged for this purpose. In addition, participants suggested coupling exposure measures with existent cohort studies (e.g., in cardiovascular disease) that collect samples and health information. Participants called for research plans to be developed in advance of the next wildfire smoke season.

Participants emphasized the need for collaboration across sectors. To collaboratively address the research needs identified throughout the discussions, they identified opportunities for partnerships between universities and researchers who work in forest health, fire science, air pollution, environmental and public health; emergency managers; clean air agencies; and the Northwest air quality communicators group. Moreover, the Northwest Fire Science Consortium is active in wildfire research and has connections with practitioners. Leveraging the expertise and partnerships across these entities could serve as an important resource for communicating health risk information to practitioners.

Participants noted the need for equal partnerships among communities and researchers in community-based participatory research. It is necessary for researchers to spend time in rural communities to work with rural populations.

Participants described a network of low-cost air monitors established in the Puget Sound region as part of the University of Washington-based study on Alzheimer’s disease and air pollution and noted the establishment of similar networks and initiatives in rural communities should be explored. They also identified the need for enhanced forecasting capabilities.

**Limitations**: While we sought a diverse representation of stakeholders at the symposium, the event was organized by University of Washington faculty and students, held on the University of Washington campus and University of Washington faculty, staff, and students (combined) were the largest stakeholder group represented. It is possible that University of Washington-based research capabilities and expertise (for example, related to exposure science and air quality), as well as University of Washington attendee interest, influenced the discussion and findings reported herein. Moreover, comments captured in the notes taken at the symposium, which were used as the data source for the qualitative content analysis reported herein, were not attributed to any individual participant. As such, we were unable to follow-up with specific individuals for further information about the nature or rationale for their comments (e.g., the reason for discussion of VOC exposure research and not research on ozone, or which specific VOCs are of primary interest). Finally, diverse interest in and needs to enhance the science related to health impacts of wildfires, including wildfire smoke, was underscored at the June 2019 National Academies of Science, Engineering and Medicine’s meeting, “Implications of the California Wildfires for Health, Communities, and Preparedness: A Workshop” [18]. Research published in the months since our symposium reflects the fact that wildfire smoke and health is a fast-moving and dynamic area of research (e.g., estimating wildfire-related exposure to ozone and PM2.5, and estimations and their associations with respiratory health) [19]. As such, the research needs presented herein should be continuously updated and interpreted as broad areas of interest to Washington practitioners and academics. Future research endeavors should be guided by the most recently available scientific evidence.

## 4. Conclusions

Additional evidence is needed to support risk assessment, risk communication, and risk management to protect communities and workers across Washington state from the growing threat of wildfire smoke. Researchers, practitioners, and funders are encouraged to use the findings from this symposium reported herein to inform future research-practice collaborations and policy- and practice-relevant research.

## Figures and Tables

**Table 1:** Participants’ organization types.

Organization	*N*	%
University of Washington (Faculty/Staff) *	21	27.6
University of Washington (Student) *	11	14.5
Academia (non-University of Washington)	2	2.6
State public health agency	5	6.6
Local public health agency	9	11.8
Air quality agency	9	11.8
Non-public health local government agency	2	2.6
Non-public health state government agency	5	6.6
Tribal government	2	2.6
Federal government	3	3.9
Non-governmental organization	5	6.6
Media	2	2.6

* Includes the organizing author team, as well as nine additional University of Washington-based world café facilitators and notetakers.

**Table 2:** Key needs by research topic.

Research Topic	Needs
Exposure	Quantify exposure by geographic distance from fire Develop a definition of a “wildfire smoke event” Understand smoke characteristics and their impact on health Determine impacts of concurrent exposure with pesticides Assess indoor and automobile cabin air
Health Risk	Assess short- and long-term health impacts to smoke and smoke component exposure among subpopulations (e.g., “healthy,” at-risk groups, workers) Assess health impacts of short-term (i.e., acute), long-term (i.e., chronic) and cumulative exposure Quantify relative risks of exposure across different air quality index categories
Risk Communication	Assess risk perceptions and their determinants Identify community assets and educational needs Evaluate the effectiveness of specific messages, communication mechanisms, and prior communication efforts for each at-risk group
Behavior Change and Interventions	Assess the effectiveness, sufficiency, and feasibility of different behaviors (e.g., staying indoors), individual-level interventions (e.g., N-95 mask use), workplace controls, and community-level interventions (e.g., clean air spaces) to protect health and well-being Describe current individual responses (e.g., behavior change and intervention uptake) during wildfire smoke events
Legal and Policy	Assess the appropriateness and feasibility of using occupational health and safety regulations to control exposure Develop science-based exposure limits for indoor and outdoor workers Determine appropriate policy solutions for liability and workers compensation Conduct health impact assessments of proposed policy changes (e.g., building code changes) Identify international policy and practice responses to wildfire smoke

## References

[1] National Interagency Fire Center. https://www.nifc.gov/fireInfo/fireInfo_statistics.html.

[2] Envitech LTD, Israel Washington State—Air Monitoring. https://fortress.wa.gov/ecy/enviwa/.

[3] De May D. (2018). How Harmful Is It to Breathe the Wildfire Smoke Blanketing Seattle? In Seattlepi.com. Seattle Post-Intelligencer. https://www.seattlepi.com/seattlenews/article/Harmful-to-breathe-the-wildfire-smoke-Seattle-13158856.php.

[4] Liu J.C., Pereira G., Uhl S.A., Bravo M.A., Bell M.L. (2015). A systematic review of the physical health impacts from non-occupational exposure to wildfire smoke. Environ. Res..

[5] Reid C.E., Brauer M., Johnston F.H., Jerrett M., Balmes J.R., Elliott C.T. (2016). Critical Review of Health Impacts of Wildfire Smoke Exposure. Environ. Health Perspect..

[6] Cascio W.E. (2018). Wildland fire smoke and human health. Sci. Total Environ..

[7] Abatzoglou J.T., Williams A.P. (2016). Impact of anthropogenic climate change on wildfire across western US forests. Proc. Natl. Acad. Sci. USA.

[8] Westerling A.L., Hidalgo H.G., Cayan D.R., Swetnam T.W. (2006). Warming and earlier spring increase western U.S. forest wildfire activity. Science.

[9] Littell J.S., Oneil E.E., McKenzie D., Hicke J.A., Lutz J.A., Norheim R.A., Elsner M.M. (2010). Forest ecosystems, disturbance and climatic change in Washington State, USA. Clim. Chang..

[10] Westerling A.L. (2016). Increasing western US forest wildfire activity: Sensitivity to changes in the timing of spring. Philos. Trans. R. Soc. B Biol. Sci..

[11] Flannigan M., Stocks B., Turetsky M., Wotton M. (2009). Impacts of climate change on fire activity and fire management in the circumboreal forest. Glob. Chang. Biol..

[12] Gillett N.P. (2004). Detecting the effect of climate change on Canadian forest fires. Geophys. Res. Lett..

[13] Spracklen D.V., Mickley L.J., Logan J.A., Hudman R.C., Yevich R., Flannigan M.D., Westerling A.L. (2009). Impacts of climate change from 2000 to 2050 on wildfire activity and carbonaceous aerosol concentrations in the western United States. J. Geophys. Res..

[14] (2007). Climate Change 2007: Impacts, Adaptation and Vulnerability. Contribution of Working Group II to the Fourth Assessment Report of the Intergovernmental Panel on Climate Change.

[15] World Café Method (2015). In The World Café. http://www.theworldcafe.com/key-concepts-resources/world-cafe-method/.

[16] Miles M.B., Michael Huberman A., Huberman M.A., Huberman P.M. (1994). Qualitative Data Analysis: An Expanded Sourcebook.

[17] Minkler M., Garcia A.P., Rubin V., Wallerson N. (2012). A Strategy for Building Healthy Communities and Promoting Health through Policy Change: A Report to the California Endowment. Policy Link. http://www.policylink.org/sites/default/files/CBPR.pdf.

[18] (2019). Implications of the California Wildfires for Health, Communities, and Preparedness: A Workshop. The National Academies of Science, Engineering and Medicine. http://www.nationalacademies.org/hmd/Activities/PublicHealth/ImplicationsoftheCaliforniaWildfiresforHealthCommunitiesandPreparedness/2019-JUNE-04.aspx.

[19] Reid C.E., Considine E.M., Watson G.L., Telesca D., Pfister G.G., Jerrett M. (2019). Associations between respiratory health and ozone and fine particulate matter during a wildfire event. Environ. Int..

